# Laryngeal Mask Airway Does Not Reduce Postoperative Nasal Bleeding Outside the Operation Room after Intranasal Surgery

**DOI:** 10.1155/2013/461023

**Published:** 2013-11-28

**Authors:** Xuyu Zhang, Xia Feng, Xiaodan Wu, Zimeng Liu, Hufei Zhang, Xinhe Liu

**Affiliations:** ^1^Department of Anesthesiology, The First Affiliated Hospital of Sun Yat-sen University, No. 58 Zhongshan 2nd Road, Guangzhou, Guangdong 510089, China; ^2^Department of Surgical Intensive Care Unit, The First Affiliated Hospital of Sun Yat-sen University, No. 58 Zhongshan 2nd Road, Guangzhou, Guangdong 510089, China

## Abstract

*Background*. The aim of this study was to detect the effect of the laryngeal mask airway (LMA) versus the endotracheal tube (ETT) on postoperative nasal bleedings in and outside the operation room (OR) after intranasal surgery. *Methods*. 134 patients undergoing elective intranasal surgeries were randomly allocated to receive LMA or ETT during general anesthesia. The incidence, episodes, and severity of nasal bleeding were evaluated in the OR and within the postoperative 24 hours in the ward. Furthermore, medical assistance and severe complications were assessed. *Results*. The overall incidence of postoperative nasal bleeding throughout the observation period was similar between the two groups. The LMA reduced nasal bleeding in the OR. However, outside the OR, the incidence of the first episode of postoperative nasal bleeding in the LMA group was higher than that in the ETT group (difference: −26.5%; 95% CI: −42.2% to −10.7%; *P* < 0.001). In the LMA group, more patients needed medical assistance (*P* = 0.029), and the number of assistance was also higher (*P* = 0.027) in the ward. No severe complications occurred during the observation period. *Conclusion*. The LMA does not alleviate nasal bleeding conditions and even increases the demands of medical service outside the OR after intranasal surgery, although it reduces epistaxis during extubation.

## 1. Introduction

In the past decades, the number of nasal and sinus surgeries has grown all over the world. It was reported that several hundred thousand surgeries were performed each year in the United States [1]. And postoperative excessive bleeding is still the common and severe complications after nasal surgeries [2, 3]. A retrospective study showed that epistaxis was the leading cause for overstay or readmission after day-case nasal procedures [4]. Different types of hemostatic agents or neotype packing had been applied to reduce the postoperative bleeding. However, these surgical techniques did not show any benefits, or some nasal packings even increased patients' pain [5–7]. Therefore, investigation of a new means to prevent postoperative nasal bleeding is of clinical importance.

The laryngeal mask airway (LMA) has been proved to be suitable for the intranasal surgery [8–11]. When compared with the tracheal intubation, the LMA has many advantages, such as minimal cardiovascular response, lack of tracheal stimulation, less sore throats [12]. Based on these advantages, several clinicians hypothesized that LMA might reduce the risk of nasal bleeding during extubation after nasal procedures. To date, however, this hypothesis had not been clearly confirmed. In addition, because most elective nasal operations are performed as day cases, it is important to control bleeding both in and outside the operation room (OR). To the best of our knowledge, no previous report has demonstrated the early and late effect of the LMA on postoperative epistaxis after nasal surgeries. Therefore, we hypothesized that the use of LMA might be an effective treatment for postoperative nasal bleeding.

The aim of this randomized, double-blinded, and controlled study was to compare the LMA with the endotracheal tube (ETT) on nasal bleeding control after the intranasal surgery.

## 2. Methods

This prospective study was approved by the institutional review board of The First Affiliated Hospital of Sun Yat-sen University, and the written consent was obtained from each patient. American Society of Anesthesiologists grade I-II, aged 18–65 years patients undergoing elective intranasal surgeries were enrolled in the study. The patients with contraindications of LMA, severe systematic diseases, body Mass Index <18 or >30 kg/m^2^, inability to communicate effectively, or allergy to any of drugs used in our study were excluded.

Patients who met the inclusion criteria were registered by using sealed envelopes with block randomization generated by SAS software and allocated to either of the two groups. Both the randomization lists and envelopes were securely stored by an independent technician. Participants and observers were masked to group assignment.

On the patient's arrival in the OR, vital signs and bispectral index were monitored. An attending doctor who was not involved in postoperative assessment opened the next randomized envelope in the sequence and assigned the patients into two groups. Group LMA: a flexible reinforced laryngeal mask airway was applied; Group ETT: a traditional endotracheal tube was applied.

Following 3 minutes of preoxygenation (10 L·min^−1^) by the face mask, propofol 2.5 mg·kg^−1^, cisatracurium 0.12 mg·kg^−1^, and fentanyl 1 *μ*g·kg^−1^ were administered to each patient to facilitate the airway device insertion. The LMA or ETT was placed and secured through the standard regimens by the experienced consultants. The mechanical ventilation was adjusted to maintain the P_ET_CO_2_ between 35 and 40 mmHg and the peak airway pressure below 20 cm H_2_O. The general anesthesia was maintained with propofol and remifentanil. During the surgery, propofol was titrated to maintain bispectral index between 40 and 60, and remifentanil was adjusted to maintain mean arterial pressure between 60 and 80 mmHg. All patients received intravenous parecoxib sodium 40 mg and tropisetron 6 mg approximately 30 minutes before the end of surgery.

The propofol and remifentanil infusion were stopped at the insertion of nasal packing (expansive sponge). The anesthesia time (from the induction of anesthesia to the discontinuation of anesthetics) was noted. The airway device was hided under a drape. An independent and well-trained observer came in and assessed the grade of nasal bleeding (0 = no bleeding; 1 = bloody secretion in oral and nasal cavity; 2 = self-limiting bleeding; 3 = conservative treatment; 4 = operative intervention) until extubation [6]. The LMA or RTT was deflated and simultaneously removed when patients opened their eyes and responded to verbal commands. This observer went out during extubation and came back to continue recording nasal bleeding conditions and severe side effects. Once the patients had achieved Steward score >4 points, they were transferred to the ward.

In the ward, the patients were requested to record the number of nasal bleeding episodes and evaluate the severity of bleeding by using a visual analog scales (VAS, scored from 0 to 100) during postoperative 24 hours. The definition of this VAS is 0 = no bleeding, 50 = moderate bleeding requiring frequent use of facial tissues, and 100 = severe or constant bleeding [5]. The observer came at 24 hour after surgery and recorded the bleeding conditions, number of medical assistance, and other severe complications.

### 2.1. Statistical Analysis

The overall incidence of nasal bleeding within the whole observation period (in and outside the OR) was set as the primary endpoint in the present study. According to the data during emergence in our previous study [13], the incidence of bleeding was preliminarily estimated as 25% and 7.5% in the ETT and LMA group, respectively. Based on a method of sample size calculation [14], 66 patients for each group were required for a chi-square test with power = 0.8 and *α* = 0.05 (2-sided). Considering the possible 5% drop outs, we decided to enroll 140 patients. Demographic and surgical data were presented as mean ± standard deviation, numbers of patients. Student's *t*-test was used to compare continuous variables between groups. The primary endpoint and other dichotomous outcomes were compared by *χ* ^2^ test or Fisher's exact test. The differences of incidence between two groups were presented as rate difference (RD) with 95% confidence interval (CI). Because of a nonnormal distribution, the episodes and intensity of bleeding were presented as median and range and were analyzed with Mann-Whitney *U* test. All data were analyzed by using SAS 9.1 software (SAS Institute, Cary, NC, USA) and SPSS 15.0 software (SPSS, Chicago, IL, USA). All reported *P* values were two-sided.

## 3. Results

**Figure 1:**
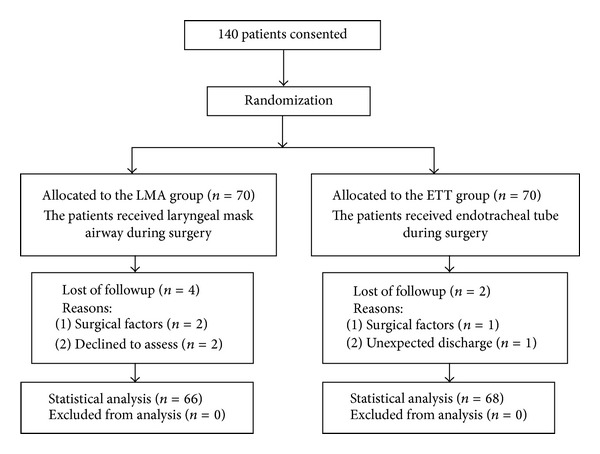
Flow diagram (progress steps of the trial: selection and randomization of elective intranasal patients to receive the laryngeal mask airway or endotracheal tube).

From Mar 2011 to September 2012, a total of 140 patients were enrolled in this study. Four patients and two patients were excluded in the LMA and ETT groups, respectively (Figure 1). 134 patients were finally included in data analysis.

Demographic and surgical characteristics were shown in Table 1. There were no clinically important differences in patients' and surgical characteristics between two groups (Table 1).

Overall, 40 of 66 (60.6%) patients in the LMA group and 36 of 68 (52.9%) patients in the ETT group experienced nasal bleeding within the whole study period. There was no significant difference between two groups (RD = −7.7%; 95% CI: −24.4% to 9.1%; *P* = 0.371).

In the OR, the incidence of nasal bleeding in the ETT group was significantly higher than that in the LMA group (RD = 18.8%, 95% CI: 5.7% to 31.9%; *P* = 0.007; Table 2). In the ETT group, the episodes and grade of nasal bleeding were also higher (Table 2). One patient and five patients needed medical interventions due to epistaxis in the LMA and ETT groups, respectively. No patients received reoperation and no severe side effects were recorded.

Outside the OR, there were no statistically significant differences in the incidence, episodes, and intensity of nasal bleeding between the two groups (Table 3).

In the LMA group, 33 patients who did not suffer epistaxis in the OR firstly experienced nasal bleeding after surgery. In the ETT group, only 16 patients firstly experienced postoperative nasal bleeding in the ward. Thus, outside the OR, the incidence of the first episode of epistaxis in the ETT group was lower than that in the LMA group (RD: −26.5%; 95% CI: −42.2% to −10.7%; *P* < 0.001; Table 3). Less patients in the ETT group needed medical assistance (RD: −15.5%; 95% CI: −28.7% to −2.3%; *P* = 0.029) and the number of assistance was also lower in the ETT group (Table 3). No participants received emergency surgery and no severe complication was observed in two groups outside the OR.

## 4. Discussion

A previous report showed that 24 of 447 (5.4%) patients receiving ambulatory nasal surgery required unexpected overnight admissions due to nasal hemorrhage in the immediate postoperative period [15]. Furthermore, the readmission rate of septal surgery was up to 14.3%, because of postoperative epistaxis [16]. And various techniques were investigated to find an effective and simple regimen for epistaxis control. As the LMA was recommended as an alternative to a traditional endotracheal tube in sinonasal surgery, we attempted to determine whether the LMA could reduce postoperative nasal bleeding after intranasal surgery.

Because of the risk of aspiration of blood into patients' upper airway, awake extubation is usually performed in sinonasal surgery. In the present study, the removal of airway devices was implemented when patients opened their eyes and responded to verbal commands. However, awake extubation is consequentially accompanied by excessive bleeding and oxyhemoglobin desaturation [8]. When compared to the ETT, the LMA does not directly stimulate the larynx. So, the LMA probably decrease postoperative bleeding by minimizing airway stimulations, cardiovascular responses and coughing during emergence [17–19]. And our results also clearly showed that, in the OR phase, the incidence, episodes, and severity of nasal bleeding in the LMA group were lower than that in the ETT group. This indicates that the LMA possesses advantages for postoperative nasal bleeding control during early recovery phase (in the OR).

In the ward, however, the use of LMA did not alleviate nasal bleeding but increase the need of medical assistance. The hemostatic superiority of LMA disappeared after the patients came back to the ward. Similarly, Jameson and colleagues reported that, in endoscopic sinus surgery, a neotype agent resulted in less nasal bleeding in the immediate postoperative period, but not in the later time [20]. And therefore this agent was just recommended to be suitable for immediate postoperative epistaxis. In our study, outside the OR, the first episode of postoperative epistaxis occurred in 16 of 68 (23.5%) patients in the ETT group, whereas 33 of 66 (50%) patients in the LMA group who did not suffer epistaxis in the OR firstly experienced nasal hemorrhage in the ward. Apparently, a sudden oronasal bleeding is distressing for both patients and their carers. Thus, more medical services were needed. We concluded that smooth emergence caused by the LMA might decrease the probability of epistaxis in the potential bleeding wounds during the emergence. In other words, the LMA perhaps prevents identification of potentially problematic situations intraoperatively. And, in the ward, resumption of the patients' daily activities may lead to the onset of bleeding. This important finding implied that the LMA might increase the number of medical assistance or revisit the hospital after intranasal surgery due to a higher risk of later nasal bleeding. For the procedures with high incidence of postoperative bleeding (e.g., septoplasty and polypectomy), the LMA seems to be an unsuitable choice. Moreover, detailed explanation and suggestion for postoperative nasal bleeding shall be given to the patients receiving LMA to reduce the anxiety and potential complications.

The previous reports about sinonasal surgery focused on the advantages of the LMA during emergence [21]. The present study investigated effect of the LMA on postoperative nasal bleeding not only in the OR but also outside the OR. We suggest that our findings may help the clinicians to choose an appropriate airway management for the patients in the sinonasal operations.

The present study had several possible limitations. Firstly, postoperative assessment was performed within 24 hours after surgery because almost all of severe epistaxis occur in the immediate postoperative period [15]. A prolonged assessment may show the long-term effect of the LMA on nasal bleeding more clearly. Secondly, we calculated the sample size based on the incidence of bleeding during extubation. But the present data showed that the overall incidences of epistaxis between two groups were closely similar. Thus, a large sample size research may detect more differences between the LMA and ETT.

In conclusion, when compared to the ETT, the LMA reduces postoperative nasal bleeding in the OR after intranasal surgery. Outside the OR, however, the LMA shows no advantages on bleeding control, but it may increase the requirements of medical service due to later nasal bleeding.

## Figures and Tables

**Table 1:** Demographic and surgical characteristics.

	LMA group *n* = 66	ETT group *n* = 68	*P* value
Age (yr)	37 ± 11	33 ± 15	0.686
Weight (kg)	56 ± 17	60 ± 14	0.724
Height (cm)	164 ± 15	169 ± 18	0.446
Sex (male/female)	40/26	35/33	0.287
ASA physical status (I/II)	60/6	57/11	0.218
Type of surgery			0.619
Sinus surgery	26	29	
Septoplasty/turbinate surgery	12	11	
Sinus + septoplasty	22	26	
Plastic surgery	4	1	
Others	2	1	
Surgery time (min)	55 ± 16	60 ± 20	0.568

The data are expressed as mean ± SD or numbers (*n*).

LMA: laryngeal mask airway; ETT: endotracheal tube.

**Table 2:** Nasal bleeding conditions in the operation room.

	LMA group *n* = 66	ETT group *n* = 68	*P* value
Incidence of bleeding	7 (10.6)	20 (29.4)	0.007
Total number of episodes	0 (0–2)	0 (0–4)	0.005
The severity of bleeding	0 (0–2)	0 (0–4)	0.004
Grade 0	59 (89.4)	48 (70.6)	
Grade 1	5 (7.6)	4 (5.9)	
Grade 2	2 (3)	12 (17.7)	
Grade 3	0	3 (5.9)	
Grade 4	0	0	
Medical interventions	1 (1.5)	5 (7.4)	0.208
Reoperation	None	None	1.000
Severe complications	None	None	1.000

The data are expressed as numbers (%) or median (range).

LMA: laryngeal mask airway; ETT: endotracheal tube.

**Table 3:** Nasal bleeding conditions outside the operation room.

	LMA group *n* = 66	ETT group *n* = 68	*P* value
Incidence of bleeding	38 (57.6)	35 (51.5)	0.478
First episode of bleeding	33 (50)	16 (23.5)	0.001
Total number of episodes	1 (0–6)	1 (0–4)	0.198
The VAS score	30 (0–100)	15 (0–100)	0.129
Medical assistance	18 (27.3)	8 (11.8)	0.029
The number of assistance	0 (0–4)	0 (0–2)	0.027
Reoperation	None	None	1.000
Severe complications	None	None	1.000

The data are expressed as numbers (%) or median (range).

LMA: laryngeal mask airway; ETT: endotracheal tube.

VAS: visual analog scales, for evaluating the severity of bleeding.
